# People with activity limitations’ perceptions of their health condition and their relationships with social participation and experienced autonomy

**DOI:** 10.1186/s12889-019-7698-9

**Published:** 2019-11-19

**Authors:** Tineke Meulenkamp, Mieke Rijken, Mieke Cardol, Anneke L. Francke, Jany Rademakers

**Affiliations:** 1 NIVEL, Netherlands Institute for Health Services Research, P.O. Box 1568, Utrecht, 3500 BN The Netherlands; 2 Department of Health and Social Care, University of Eastern Finland, Kuopio, Finland; 3 Rotterdam University of Applied Sciences, Research Centre Innovations in Care, Rotterdam, The Netherlands; 4 Amsterdam UMC, Vrije Universiteit Amsterdam, Amsterdam Public Health Research Institute, Amsterdam, The Netherlands; 5 Department of Family Medicine, Care and Public Health Research Institute (CAPHRI), Maastricht University, Maastricht, The Netherlands

**Keywords:** Disability, Illness perceptions, Self-regulation, Social participation, Autonomy

## Abstract

**Background:**

People with activity limitations participate less in society, which may be due to both societal barriers and personal factors. The aim of this study was to examine the role of one specific personal factor, namely the perceptions that people have of their health condition. We hypothesized that perceptions of more personal control and less negative consequences increase the likelihood of participation in social activities and of experiencing autonomy in participation.

**Methods:**

Survey data of 1681 people with activity limitations participating in a Dutch nationwide panel-study were analyzed by means of logistic and linear regression analyses. Perceptions of the health condition were assessed with the revised Illness Perception Questionnaire (IPQ-R). Social participation was operationalized as doing volunteer work, participating in club activities and meeting friends. Two scales of the Impact on Participation and Autonomy questionnaire were used to assess experienced autonomy regarding participation.

**Results:**

People who perceived more personal control over their health condition were more likely to participate in volunteer work (OR = 1.36) and club activities (OR = 1.35). People who believed their condition to be long-lasting were also more likely to do volunteer work (OR = 1.34), whereas people who reported a better understanding of their condition were more likely to frequently meet friends (OR = 1.19). Perceptions of the health condition explained 14% of the variance in experienced autonomy in participation, in addition to the severity of participants’ activity limitations and their age, gender and education level. Especially a belief in more serious consequences, a perception of a long-lasting and less controllable condition, a perception of less understanding of the condition and a greater perceived impact on the emotional state were associated with experiencing less autonomy in participation.

**Conclusions:**

People with activity limitations who experience less control over their condition participate less in volunteer work and club activities than people who experience more control. Perceptions of the health condition are just as important to explain differences in participation as the severity of people’s activity limitations and their socio-demographic characteristics. Health and social care professionals should pay attention to people’s perceptions, to help people with activity limitations to participate according to their needs, circumstances, and preferences.

## Background

Participation in society is considered one of the key components of human functioning [1], as it contributes to wellbeing and fulfillment of personal goals [2–4]. As such, participation in society has been recognized as a universal right, also for people with disabilities [5], i.e. people who have activity limitations due to diseases, disorders or injuries. Nevertheless, numerous studies have shown that people with activity limitations participate less in work and social life than people without such limitations [6–8], and many people with activity limitations also express a desire to participate more [9]. Moreover, these people often feel restricted to make their own choices regarding participation [10].

**Fig. 1:**
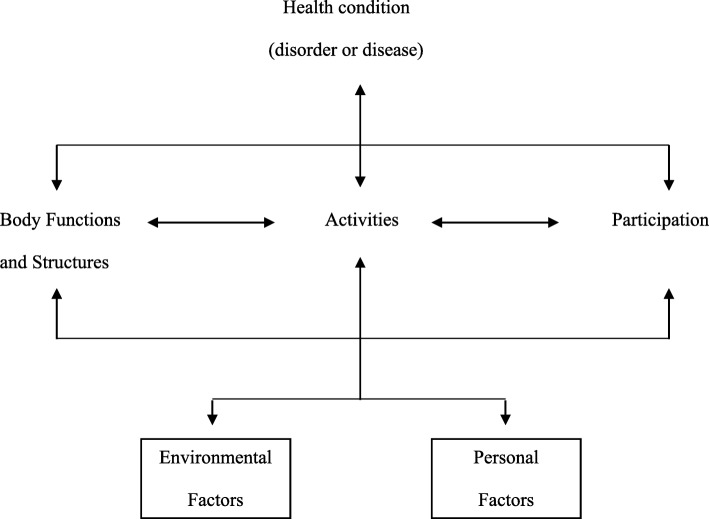
The ICF Model (Source: World Health Organization. Towards a Common Language For Functioning, Disability and Health: ICF The International Classification of Functioning, Disability and Health. Geneva: WHO, 2002)

As has been recognized by the biopsychosocial model underlying the International Classification of Functioning, Disability and Health (ICF) [1] (Fig. 1), the participation problems experienced by people with activity limitations are not simply caused by their activity limitations or the underlying health condition (disease, disorder or injury), but result from a mismatch between their needs and preferences on the one hand and societal norms, contexts and characteristics on the other hand. While it is beyond doubt that societal barriers such as poor accessibility of buildings and public transport or prejudices of other people impede participation of people with activity limitations [11], personal factors may also play a role. In this study we aimed to gain more insight in the role of personal factors by exploring how the perceptions that people with activity limitations have of their health condition relate to their social participation. Personal perceptions of one’s health condition relate to various health and illness behaviours [12, 13]. Given this relationship with behavioral outcomes, we reasoned that such perceptions might also play a role in explaining differences in the extent to which people participate in social activities, as social participation can be considered a behavioral outcome as well. Having a better understanding of people’s perceptions of their health condition and how these impact their social participation could help to develop policies and interventions to reduce the gap between the needs and preferences of people with activity limitations regarding participation and their actual participation in society.

To study the perceptions of people with activity limitations of their health condition, we started from the Common Sense Model of self-regulation (CSM) [12], as this model provides a theoretical framework to explain behavioral responses to health threats. As people with activity limitations are continuously challenged to cope with daily difficulties arising from their functional status, we assume that their condition could be considered a health threat. According to the CSM, people develop their own representations or perceptions of a health threat, which in turn determine how they cope with it, adapt to it and make behavioural choices. These perceptions are built on information from health care professionals, important others or (social) media and previous experiences (own experiences or those of others) as well as social or cultural factors, and generally include perceptions of symptoms that relate to the health condition, what caused the condition, how long it will last, its consequences and controllability. It also includes perceptions about the extent to which a person is emotionally affected by the health condition. A vast amount of research has shown that all these perceptions differ among people, also among people with similar conditions, and contribute to explain differences among people in health and illness behaviours and aspects of quality of life [14–17]. Other studies have demonstrated perceptions of one’s health condition to impact the participation in paid work [18, 19].

In this study we focused on the role of peoples’ perceptions of their health condition in relation to participation in unpaid (volunteer) work and social activities, as these are important domains of participation for older people [20], who constitute the majority of the population of people with activity limitations [21]. Besides on the actual participation in volunteer work and social activities, we focused on the autonomy that people with activity limitations experience with regard to their participation. This refers to the extent to which people feel they could live their lives as they wish and make their own choices in this respect. Autonomy can be considered vital for participation of people with activity limitations [22]. Our main research question was:

Do perceptions of people with activity limitations about their health condition relate to their actual participation in volunteer work and social activities, and to their autonomy in participation?

Based on theoretical insights about the importance of the perceived severity of a health threat and personal control beliefs for health behavior [23], as well as insights from empirical studies on the role of perceptions of the health condition in the work domain [19], we hypothesized in particular that perceptions of more personal control and less negative consequences could increase the likelihood of participation in volunteer work and social activities, and of experiencing autonomy in participation, in addition to socio-demographic characteristics such as age, gender and education level and the severity of the activity limitations.

## Methods

### Study sample and data collection

The study sample was selected from the National Panel of people with Chronic illness or Disability (NPCD), a nationwide prospective panel-study in the Netherlands [9]. This panel-study focused on developments in the quality of life and social participation of people with chronic illness or long-term activity limitations and their perceptions and use of health and social care. The panel consisted of approximately 4000 people who were recruited from general practices (random samples of general practices drawn from the Dutch registration of general practices) and national population surveys. To be included in the panel-study, people had to have a diagnosis of one or more somatic chronic disease(s) and/or to experience activity limitations (assessed by a self-report screening questionnaire [24]) to some degree. Additional inclusion criteria were: aged 15 or older, not being institutionalized, not being terminally ill (a life expectancy of at least 6 months according to their primary care physician) and sufficient mastery of the Dutch language.

Panel members provided information about their social participation by an annual survey in October, as part of a national monitoring study [9]. For the current study, we used the social participation data collected in October 2012 (response rate 85%) and collected additional data about participants’ perceptions of their health condition in April 2012 (response rate 84%). Furthermore, for the purpose of this study we only included panel members with activity limitations (thus excluding panel members with a chronic disease who did not experience activity limitations) and who responded to both questionnaires (*N* = 1861).

### Measures

#### Perceptions of the health condition

Perceptions of the health condition were assessed with the Illness Perception Questionnaire-Revised (IPQ-R) [25]. To ensure that the items were appropriate for all participants with activity limitations, irrespective of whether their activity limitations were caused by diseases, disorders or injuries, we replaced the word “illness” by “condition” in all items. We used eight scales of the IPQ-R: identity, timeline (acute/chronic and cyclical), consequences, personal control and treatment control, coherence and emotional representations (Table 1). Before answering the IPQ-R questions, participants were asked to fill in the chronic condition or impairment they had; participants with multiple health conditions were instructed to answer the IPQ-R questions with reference to the condition or impairment that had the most impact on their lives. Cronbach’s alpha coefficients of the IPQ-R scales in our study ranged from .70 (Treatment control) to .91 (Emotional representations).

**Table 1:** People with activity limitations’ perceptions of their health condition, their social participation and experienced autonomy; descriptive statistics

Perceptions of the health condition		N	M	SD
Identity	14 common symptoms, occurrence and relation to their health condition. Higher scores indicate a belief that more symptoms relate to the condition (range 0–14).	1526	5.2	2.7
Timeline acute/chronic	6 items, e.g. “My condition is likely to be permanent rather than temporary”. Higher scores indicate a belief in a more chronic timeline (range 1–5).	1641	4.5	0.6
Timeline cyclical	4 items, e.g. “My condition is very unpredictable”. Higher scores indicate a belief in a more cyclical timeline (unpredictability) (range 1–5).	1629	3.2	0.9
Consequences	6 items, e.g. “My condition has major consequences on my life”. Higher scores indicate a perception of more serious consequences (range 1–5).	1642	3.6	0.8
Personal control	6 items, e.g. “I have the power to influence my condition”. Higher scores indicate a perception of more personal control (range 1–5).	1639	2.9	0.8
Treatment control	5 items, e.g. “There is nothing which can help my condition”. Higher scores indicate a perception of more control by means of medical treatment (range 1–5).	1629	2.6	0.7
Coherence	5 items, e.g. “My condition doesn’t make any sense to me”. Higher scores indicate a better perceived understanding of the condition (range 1–5).	1626	3.7	0.8
Emotional representations	6 items, e.g. “When I think about my condition I get upset”. Higher scores indicate a greater perceived impact on the emotional state (range 1–5).	1634	2.7	0.9
Social participation		N	%	
Doing volunteer work	1 item, “Do you perform volunteer services? (e.g. for a sports club, church, school, political party)?”	1592	26	
Performing club activities weekly	1 item, “How often do you participate in the following club activities: (1) sports in a club, (2) dance/music/drama/hobby club?”	1582	31	
Meeting friends weekly	1 item, “How often do you meet friends or good acquaintances? (we mean meetings with people who do not live in your house and with whom you have at least a short conversation, not just a greeting)”	1606	38	
Autonomy		N	mean	SD
Autonomy outdoors	5 items, e.g. “My chances of using leisure time the way I want to are …” (very good to very poor; range 0–4)	1656	1.8	0.8
Autonomy in social life and relationships	7 items, e.g. “The quality of my relationships with people who are close to me are …” (very good to very poor; range 0–4)	1625	1.3	0.6

#### Social participation

Three domains of social participation were included: doing volunteer work, performing club activities weekly and meeting friends or good acquaintances weekly. Each domain was assessed by a single item developed together with stakeholders and previously tested within the framework of the national monitoring study [9] (Table 1). The scores of each item were dichotomized to obtain an indicator of whether a respondent participated (to some extent) in the specific domain. In addition, autonomy in participation was assessed by two scales of the Impact on Participation and Autonomy (IPA) questionnaire: Autonomy outdoors and Autonomy in social life and social relationships [26]. Higher scores on the scales indicate that people experience less autonomy in their participation, i.e. more restrictions in living the way they want to live and less choice in taking part in activities that are important to them. Cronbach’s alpha coefficients of the two scales reported by the original authors [26] were .81 (Autonomy outdoors) and .86 (Autonomy in social life and relationships); in our study Cronbach’s alpha of both scales was .85.

#### Control variables

Participants’ sex, age, education level, living situation and severity of their activity limitations were included as control variables. Education level was categorized in low (no education, primary school or vocational training), medium (secondary or vocational education) and high (professional higher education or university). Living situation was dichotomized: living with or without a partner or spouse. The severity of participants’ activity limitations was assessed by a self-report validated questionnaire comprising 24 items referring to three dimensions: motor limitations, visual and hearing impairments [24]. According to the official guidelines [24], participants’ severity of activity limitations was primarily based on the severity of their motor limitations. Mild limitations refer to experiencing difficulty in the execution of one or more Instrumental Activities of Daily Living (IADL), such as preparing meals or doing household activities. Moderate limitations refer to difficulties in executing various activities, not only IADL, but also mobility-related. Severe limitations are defined as being unable to perform at least one activity in the IADL or ADL domain independently, i.e., without assistance. In case participants with mild limitations report moderate or severe visual or hearing impairments, the severity of their activity limitations is raised to respectively moderate or severe.

For descriptive purposes only, we also provide information about the type of chronic disease(s) the participants had been diagnosed with. The diagnoses of the chronic disease(s) were registered by the primary care physicians with permission of the panel members. These diagnoses included somatic diseases or disorders which were defined as chronic (i.e., not (completely) curable, with a life-long duration and burdening health care or self-management) or not chronic by definition but with symptoms known by the primary care physician for at least 1 year.

### Statistical analysis

Univariate analyses were performed to describe the characteristics of the sample and the mean scores and standard deviations of the total sample on the IPQ-R dimensions and the participation and autonomy measures. Pearson correlations were computed to examine whether the eight IPQ-R dimensions correlated in an understandable way and as such provided a coherent representation of participants’ health perception.

To answer the research question, we conducted regression analyses of two models, with participation (in volunteer work, club activities and meeting friends) or autonomy in participation outdoors and in social life/relationships as dependent variables. In a first model participants’ socio-demographic characteristics and severity of their activity limitations were included as independent variables. In a second model participants’ perceptions of their health condition were added as independent variables. In this way we could assess the value of participants’ perceptions of the health condition to explain their social participation or experienced autonomy in addition to their socio-demographic characteristics and severity of their activity limitations. With the actual participation measures being the dependent (dichotomous) variable, logistic regression analyses were conducted and the change in Nagelkerke R^2^ (between model 1 and 2) was considered as a measure for the explanatory contribution of the perceptions of the health condition. Odds ratios (OR) and confidence intervals (CI) regarding the independent variables in the model are presented. We also estimated average predicted probabilities based on the scores in our sample (marginal effect), to provide some insight in the effect of different scores on significant perceptions on the participation outcomes. In the case of the experienced autonomy scales being the dependent (continuous) variables, linear regression analyses were conducted and the change in Adjusted R^2^ (between model 1 and 2) is considered as a measure of the contribution of the perceptions of the health condition to the total variance explained by the model. Betas are presented to give an indication of the relative effect of each independent variable included in the model. Significance levels were set at *P* < .05. All analyses were performed using Stata 13.1.

## Results

### Sample characteristics

Of the total sample of 1861 people, 65% were women. The mean age was 68.6 years (SD 13.3 years). More than a third (38%) had a low education level; 43% a medium and 18% a high level of education. Regarding the severity of their activity limitations, 23% had mild, 53% moderate and 24% severe limitations.

For a third of the sample (33%), information about whether or not they had been diagnosed with a chronic disease was not available (data not provided by the primary care physician because of refusal of the participant or the primary care physician) and in 1% no chronic diseases had been diagnosed. Cardiovascular disease (26%) and musculoskeletal disorders (25%) were most prevalent; followed by COPD/asthma (16%), diabetes mellitus (14%), neurological diseases (10%), cancer (6%) and digestive diseases (5%). In 21% of the sample (also) another chronic disease (not already mentioned) had been diagnosed.

### Perceptions of the health condition

Respondents related on average five symptoms to their health condition (Table 1). Pain and fatigue were most frequently reported. In addition, respondents considered their condition as chronic (M = 4.5), quite unpredictable (M = 3.2), with relatively many consequences (M = 3.6). They perceived medium levels of personal control (M = 2.9) and control by medical intervention (M = 2.6). Furthermore, on average they believed they understood their health condition quite well (M = 3.7). The mean score on the emotional impact scale was 2.7, indicating that most participants experienced a moderate emotional impact of their health condition.

Pearson correlations (Table 2) show that the various dimensions of participants’ health perception correlated in a logical way. For instance, perceiving a better understanding of one’s health condition (coherence) related to experiencing less emotional impact of the condition, and perceiving more serious consequences of one’s condition related to more emotional impact.

**Table 2:** Perceptions of the health condition, Pearson’s correlations (*N* = 1484)

	Identity	Timeline - chronic	Timeline - cyclical	Consequences	Personal control	Treatment control	Coherence
Identity							
Timeline - chronic	0.10*						
Timeline - cyclical	0.24*	0.01					
Consequences	0.36*	0.36*	0.09*				
Personal control	−0.04	−0.15*	0.20*	−0.17*			
Treatment control	−0.07*	−0.29*	0.12*	− 0.30*	0.43*		
Coherence	−0.18*	0.16*	−0.22*	− 0.16*	0.09*	0.03	
Emotional representations	0.30*	0.01	0.24*	0.44*	−0.12*	−0.09*	− 0.49*

*P < .01

### Social participation and autonomy

About a quarter (26%) of the respondents participated in volunteer work, 31% performed club activities weekly and 38% met friends or good acquaintances weekly (Table 1). On average, respondents experienced a relatively high level of autonomy in participating in activities outdoors (M = 1.8) and with regard to their social life and relationships (M = 1.3).

### Associations between perceptions of the health condition and social participation

Respondents’ perceptions of their health condition contributed to the prediction of their actual (non) participation in several domains, given that the model including these perceptions had a better fit than the first model with only the control variables included as independent variables (Table 3). Perceptions of a longer lasting condition (OR = 1.34) and more personal control (OR = 1.36) increased the likelihood of people with activity limitations to participate in volunteer work. For a relatively low level of personal control (score 2) the model predicted a chance of 22% (95%-CI: 18–25%) to do volunteer work; for a higher level of personal control (score 4) the model predicted a chance of 33% (95%-CI: 28–38%). More perceived personal control was also associated with participation in club activities (OR = 1.35). For a low level of personal control (score 2) the model predicted a chance of 26% (95%-CI: 22–29%) to participate in club activities, and a chance of 38% (95%-CI: 33–43%) among people with a higher level of personal control (score 4). A perception of better understanding the condition was associated with a greater chance of meeting friends (OR = 1.19). For people who perceived less understanding of their condition (score 2) the model predicted a chance of 31% (95%-CI: 24–38%) to meet friends weekly, whereas in people who believed they understood their condition relatively well (score 4) the chance was 39% (95%-CI: 36–42%).

**Table 3:** Participation in volunteer work, club activities and social contact predicted by perceptions of the health condition and control variables; odd ratios (OR) and 95%-confidence intervals (CI)

	Participation in volunteer work				Performing club activities weekly				Meeting friends or good acquaintances weekly			
	Model 1# (*N* = 1582)		Model 2# (*N* = 1425)		Model 1# (*N* = 1582)		Model 2# (*N* = 1422)		Model 1# (*N* = 1606)		Model 2# (*N* = 1437)	
	OR	CI	OR	CI	OR	CI	OR	CI	OR	CI	OR	CI
Sex (female)	0.88	0.69–1.12	0.86	0.66–1.12	**1.71**	**1.34–2.17**	**1.61**	**1.24–2.09**	**1.27**	**1.02–1.59**	1.26	0.99–1.60
Age	**0.98**	**0.97–0.99**	**0.98**	**0.97–0.99**	0.99	0.98–1.00	0.99	0.98–1.00	1.00	0.99–1.00	1.00	0.99–1.01
Education (ref: low)												
Medium	1.19	0.91–1.55	1.19	0.89–1.58	1.24	0.97–1.59	1.25	0.96–1.63	0.81	0.64–1.01	0.82	0.64–1.05
High	**2.03**	**1.49–2.77**	**1.96**	**1.40–2.73**	**1.75**	**1.29–2.38**	**1.87**	**1.35–2.59**	0.80	0.60–1.08	0.85	0.62–1.16
Living situation: married / cohabiting	0.99	0.77–1.27	1.07	0.82–1.39	1.01	0.80–1.27	1.04	0.81–1.34	**0.68**	**0.55–0.84**	**0.70**	**0.55–0.88**
Activity limitations (ref: light)												
Moderate	0.77	0.58–1.01	0.87	0.64–1.19	**0.62**	**0.47–0.81**	0.80	0.59–1.08	**0.77**	**0.60–1.00**	0.90	0.67–1.19
Severe	**0.49**	**0.34–0.70**	**0.65**	**0.43–0.98**	**0.57**	**0.41–0.78**	0.83	0.57–1.22	**0.66**	**0.49–0.90**	0.74	0.51–1.07
Identity			0.98	0.93–1.03			0.98	0.94–1.04			0.99	0.95–1.04
Timeline chronic			**1.34**	**1.05–1.71**			1.03	0.83–1.28			1.08	0.88–1.33
Timeline cyclical			0.91	0.78–1.05			1.02	0.88–1.18			1.07	0.93–1.22
Consequences			0.90	0.73–1.12			0.85	0.69–1.04			0.89	0.74–1.08
Personal control			**1.36**	**1.12–1.64**			**1.35**	**1.13–1.62**			1.05	0.89–1.23
Treatment control			1.04	0.85–1.29			1.09	0.89–1.33			1.13	0.94–1.37
Coherence			0.93	0.77–1.12			1.04	0.87–1.24			**1.19**	**1.01–1.41**
Emotional representations			0.88	0.74–1.05			0.97	0.82–1.15			0.94	0.81–1.10
	Nagelkerke R^2^ 0.06		Nagelkerke R^2^ 0.08		Nagelkerke R^2^ 0.05		Nagelkerke R^2^ 0.07		Nagelkerke R^2^ 0.02		Nagelkerke R^2^ 0.04	

# significant effects (*P* < .05) are shown in bold

### Associations between perceptions of the health condition and autonomy in participation

Regarding the experienced autonomy in participation, the second model including perceptions of the health condition explained an additional 14% of the variance (Table 4). All perception dimensions appeared to be associated with experiencing autonomy in participation, except perceptions of control by medical intervention and of a cyclical timeline. The perception of the consequences of the condition had the strongest effects on experienced autonomy in participation in activities outdoors (β = .23) and in social life and relationships (β = .20).

**Table 4:** Experienced autonomy in participation predicted by perceptions of the health condition and control variables; Betas (β) and *P*-values (*P*)

	Autonomy outdoors: experienced limitations				Autonomy in social life and relationships: experienced limitations			
	Model 1# (*N* = 1656)		Model 2# (*N* = 1465)		Model 1# (*N* = 1625)		Model 2# (*N* = 1437)	
	β	*P*	β	*P*	β	*P*	β	*P*
Sex (female)	−.03	.24	−.02	.39	**−.09**	**<.001**	**−.10**	**<.001**
Age	.03	.27	**.09**	**<.001**	−.02	.45	**.03**	**.20**
Education (ref: low)								
Medium	−.04	.12	.01	.75	**−.12**	**<.001**	**−.08**	**.001**
High	−.01	.53	.01	.72	**−.14**	**<.001**	**−.12**	**<.001**
Living situation: married / cohabiting	−.04	.10	**−.05**	**.02**	**−.08**	**.001**	**−.08**	**<.001**
Activity limitations (ref: light)								
Moderate	**.37**	**<.001**	**.24**	**<.001**	**.22**	**<.001**	**.10**	**.001**
Severe	**.59**	**<.001**	**.38**	**<.001**	**.36**	**<.001**	**.18**	**<.001**
Identity			**.12**	**<.001**			**.14**	**<.001**
Timeline chronic			**−.06**	**.01**			**−.10**	**<.001**
Timeline cyclical			−.02	.49			.01	0.79
Consequences			**.23**	**<.001**			**.20**	**<.001**
Personal control			**−.10**	**<.001**			**−.10**	**<.001**
Treatment control			−.00	.99			.04	.10
Coherence			**−.08**	**.001**			**−.11**	**<.001**
Emotional representations			**.14**	**<.001**			**.12**	**<.001**
	Adj R^2^	P	Adj R^2^	P	Adj R^2^	P	Adj R^2^	P
	.23	<.01	.37	<.01	.12	<.01	.26	<.01

# significant effects (*P* < .05) are shown in bold

## Discussion

The present study examined the extent to which perceptions of the health condition of people with activity limitations determine their actual social participation and experienced autonomy in participation. We hypothesized that perceptions of personal control and of negative consequences in particular to be important determinants, which was partly confirmed by our results. In our sample we found 10% more chance to participate in volunteer work and to be engaged in club activities among people who perceive more personal control over their health condition. The role of the perceived consequences was less straightforward: a perception of more serious consequences of the health condition was not a significant predictor of actual social participation, but appeared to be the most important predictor (of all perception dimensions) of experienced autonomy in participation. Other dimensions of people’s perception of their health condition also appeared to be significant predictors of social participation or autonomy in participation. A perception of a longer lasting duration of the condition increased the chance of participating as a volunteer. In addition, a more coherent understanding of the health condition increased the chance of frequently meeting friends. With regard to experiencing autonomy in participation, almost all perception dimensions appeared to be significant predictors.

The results of our study are consistent with other studies that have shown that people’s evaluation of their situation relates to their autonomy in participation [10, 27]. More specifically, our results indicate that perceptions of one’s health condition matter when it comes to social participation and autonomy in participation. However, certain perceptions appear more or less important, dependent on the specific domain of social participation at stake. For instance, experiencing personal control over one’s health condition appeared to be important for doing volunteer work and engaging in club activities but not for meeting friends. As volunteer work and club activities usually require regular attendance, it may be that people who perceive less control over their health condition have more reservations to participate in such activities. In our study we also found that perceiving more negative consequences of one’s health condition did not relate to a lower chance of social participation, in contrast with previous studies on participation in (paid) work [18]. There may be a similar explanation for this difference as mentioned here above: social participation will generally be considered less binding than participating in paid work, which makes it more easy for people who perceive serious consequences of their health condition to decide whether or not to participate in social activities each time, dependent on how they feel. Another explanation is that the severity of people’s activity limitations itself already accounted for a large part of the (co-)variance in social participation. Further research is needed to examine the role of specific perceptions of one’s health condition for various participation domains in more detail.

### Methodological considerations

Although participants’ perceptions of their health condition were assessed 6 months prior to the assessment of their social participation and experienced autonomy, causal conclusions cannot be drawn, as we lacked multiple measurements of key variables, in particular the perceptions of the health condition. Therefore, we do not know whether people’s perceptions of their health condition are antecedents of social participation, or caused by participation experiences. Theoretical models of (health) behavior assume that cognitions and emotions are important determinants of behavior, but the cyclical nature of the relationships among these concepts is also emphasized. In addition, as perceptions of one’s health condition are shaped by everyday social interactions and past experiences [28], it seems likely that positive experiences with participation also contribute to perceptions of more control and less negative consequences of the health condition. Future research on the underlying processes is needed to gain a better understanding of the causal relationships between people’s perceptions of their health condition and social participation.

Furthermore, our sample consisted of people with activity limitations, who were very heterogeneous with regard to the cause(s) of their limitations. In this respect our study differs from most studies on illness perceptions, which usually focus on people’s perceptions of a specific disease or condition (e.g. [14–17]). Besides, some participants filled in more than one health condition for which they answered the perception questions. This should be taken into account, when comparing our findings with those of condition-specific studies [29]. Considering that the focus of our study was not on what shapes people’s perceptions of their health condition but on whether these perceptions relate to social participation, we believe that our assessment approach can be justified.

As the severity of people’s activity limitations impacts on both the perceptions of their health condition and their participation, we estimated the effects of the perception dimensions on social participation and experienced autonomy by controlling for the severity of the activity limitations of the participants. The severity of participants’ activity limitations were however assessed at inclusion in the panel-study and could have changed since then. This may imply that the correction of the estimates for activity limitation severity may have been suboptimal.

### Implications for clinical practice

Despite the limitations of this study, our results indicate that insight into the perceptions that people hold about their health condition can help understand social participation and experienced problems with participation. Therefore, it is important that health and social care professionals explore patients’ or clients’ perceptions of their health condition when discussing their participation options and restrictions. They need to be aware of the relevance of discussing the perceptions of the health condition of the patient or client, to make sure that the support they provide is tailored to a person’s individual needs, contexts and preferences. In addition, when patients or clients hold perceptions that may be restrictive, for instance, when their perceived lack of control or their view of their health condition prevents them from participating, care professionals could target these perceptions. Several studies have shown that addressing people’ perceptions of their health condition by a person-centered cognitive-behavioral approach could reduce invalidating perceptions, improve coping skills and result in better health and work outcomes [19]. Considering this, it seems likely that targeting people with activity limitations’ perceptions of their health condition could help them to participate in social activities and relationships in accordance with their personal circumstances, needs and preferences. However, intervention studies are needed to develop effective approaches that could be applied by professionals working with people with activity limitations in health and social care.

## Conclusions

People with activity limitations who experience less control over their health condition participate less in volunteer work and club activities than people who experience more control. Perceptions of the health condition may be just as important to explain differences in social participation as the severity of activity limitations and socio-demographic factors such as age, gender and education level. Health and social care professionals should pay attention to people’s perceptions, to help people with activity limitations to participate according to their needs, circumstances and preferences.

## Data Availability

The data sets used and/or analysed during the current study are available from the corresponding author upon reasonable request.

## Abbreviations

CI: Confidence interval
CSM: Common Sense Model of self-regulation
IADL: Instrumental Activities of Daily Living
ICF: International Classification of Functioning, Disability and Health
IPA: Impact on Participation and Autonomy questionnaire
IPQ-R: Illness Perception Questionnaire-Revised
NPCD: National Panel of people with Chronic illness or Disability
OR: Odds ratio
